# Delayed and limited administration of the JAKinib tofacitinib mitigates chronic DSS-induced colitis

**DOI:** 10.3389/fimmu.2023.1179311

**Published:** 2023-05-19

**Authors:** Rishav Seal, Lara S. U. Schwab, Cristina M. Chiarolla, Nadine Hundhausen, Georg Heinrich Klose, Simone Reu-Hofer, Andreas Rosenwald, Johannes Wiest, Friederike Berberich-Siebelt

**Affiliations:** ^1^ Institute of Pathology, Julius-Maximilians-University Würzburg, Würzburg, Germany; ^2^ Comprehensive Cancer Centre Mainfranken, Julius-Maximilians-University Würzburg, Würzburg, Germany; ^3^ Institute of Pharmacy and Food Chemistry, Julius-Maximilians-University Würzburg, Würzburg, Germany

**Keywords:** anti-inflammatory cytokines, AOM/DSS, pro-inflammatory cytokines, effector Treg (eTreg), chronic IBD model, JAK inhibitor, tofacitinib, treatment regimens

## Abstract

In inflammatory bowel disease, dysregulated T cells express pro-inflammatory cytokines. Using a chronic azoxymethane (AOM)/dextran sulfate sodium (DSS)-induced colitis model resembling ulcerative colitis, we evaluated whether and when treatment with the Janus kinase (JAK) inhibitor tofacitinib could be curative. Comparing the treatment with two and three cycles of tofacitinib medication in drinking water – intermittently with DSS induction – revealed that two cycles were not only sufficient but also superior over the 3-x regimen. The two cycles of the 2-x protocol paralleled the second and third cycles of the longer protocol. T cells were less able to express interferon gamma (IFN-γ) and the serum levels of IFN-γ, interleukin (IL)-2, IL-6, IL-17, and tumor necrosis factor (TNF) were significantly reduced in sera, while those of IL-10 and IL-22 increased under the 2-x protocol. Likewise, the frequency and effector phenotype of regulatory T cells (Tregs) increased. This was accompanied by normal weight gain, controlled clinical scores, and restored stool consistency. The general and histologic appearance of the colons revealed healing and tissue intactness. Importantly, two phases of tofacitinib medication completely prevented AOM-incited pseudopolyps and the hyper-proliferation of epithelia, which was in contrast to the 3-x regimen. This implies that the initial IBD-induced cytokine expression is not necessarily harmful as long as inflammatory signaling can later be suppressed and that time-restricted treatment allows for anti-inflammatory and tissue-healing cytokine activities.

## Graphical Abstract

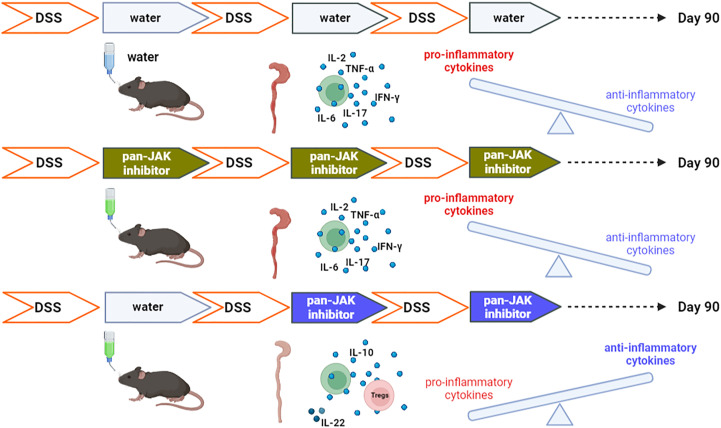

## 1 Introduction

Intestinal barrier dysfunction can cause and contribute to inflammatory bowel diseases (IBDs), in particular Crohn’s disease (CD) and ulcerative colitis (UC) (1–3). Damage to the lining of the intestine and deeper layers triggers an immune response directed against components of the gut microflora and food antigens (4, 5). Macrophages, dendritic cells, neutrophils, natural killer (NK) cells, innate lymphoid cells, and T cells of the mucosa initiate and drive the inflammatory reaction.

IBDs usually progress in a chronic, relapsing–remitting manner. CD-typical inflammation, characterized by transmural inflammation, fibrosis, and granulomatous reaction, can involve different segments of the digestive tract, most commonly the terminal ileum. UC, in contrast, is typically restricted to the colon, mucosa, and submucosa. In addition to inflammation of the (gastro-) intestinal tract, patients acquire extra-intestinal manifestations and are at risk of developing colitis-associated cancer (CAC).

Multiple aspects of IBD are controlled by cytokines (6). Activated immune cells elevate the levels of tumor necrosis factor (TNF), interleukin (IL)-1β, express interferon gamma (IFN-γ), and other pro-inflammatory cytokines, whereas the inhibitory cytokines IL-10 and TGF-β that are found in Peyer’s patches, mesenteric lymph nodes (mLNs), and lamina propria (LP) are part of the T-cell tolerance mechanisms in the intestine (7). In addition, pro-inflammatory cytokines, especially TNF and IL-6, are critical tumor promotors that trigger the development of CACs (8). Another way of distinguishing between cytokines is that pro-apoptotic cytokines promote the demise of epithelial cells and barrier disruption, while pro-survival cytokines such as IL-6 and IL-22 induce mucosal healing and improve intestinal barrier function. However, such pro-survival cytokines are also tumorigenic and facilitate the development of CAC (6, 9).

CD and UC share important end-stage effector pathways of intestinal injury, mediated by active cross-talk between immune and non-immune cells and the production of TNF, IL-1β, and IL-6 (6). Here, anti-TNF indeed represents an advance in IBD therapy in comparison with broad immune suppressive agents (10, 11). However, not all patients respond to TNF antagonists or can acquire resistance to these biologicals by developing antibodies. Single cytokine blockades—besides anti-TNF—were not successful; hence, an alternative approach is to inhibit several cytokines simultaneously by targeting common upstream signaling events. The use of Janus kinase (JAK) inhibitors such as tofacitinib represents one means of doing so.

JAKs are central intracellular mediators of cytokine signaling and their blockade can interfere with more than 50 cytokines (12–14). Normally, the binding of cytokines to their receptors leads to specific JAK family member recruitment and phosphorylation events, which in turn translocates certain Signal Transducer and Activator of Transcription proteins (STATs) to the nucleus and induce gene transcription (13, 14).

Tofacitinib is a small-molecule, ATP-competitive, reversible, and selective JAK inhibitor (JAKinib), predominantly affecting JAK3, with additional binding affinity for JAK1 and JAK2. Presently, tofacitinib is regarded as a pan-JAK inhibitor that can significantly restrict cytokine signaling (15, 16). Phase 2 and 3 clinical studies have revealed that therapy with tofacitinib induces high remission rates in UC patients compared with placebo (17). Meanwhile, it has been approved as a second-line option for the treatment of UC to be used in the event of the failure of conventional disease-modifying drugs. Nevertheless, tofacitinib—like all immunomodulatory agents (18)—incites side effects. As cytokines are involved in not only inflammation but also tissue healing, the timing and duration of treatment must influence the outcome. To evaluate such influences, we administered tofacitinib to IBD-induced mice at different disease time points in two or three cycles.

Dextran sulfate sodium (DSS)-induced colitis, i.e., a chemically induced intestinal inflammation model, morphologically and symptomatically resembles epithelial damage in UC (19, 20). Upon pre-treatment with the procarcinogen azoxymethane (AOM), DSS synergizes in promoting CAC (21). Here, repeated cycles of DSS induce chronic IBD—resembling relapsing–remitting IBD in patients—which manifests as diarrhea and bloody feces, shortened colons with increased weight and wall thickness, and dysplasia. To highlight potential benefit (which outweighs the side effects) of tofacitinib for mucosal healing, we tested tofacitinib in the chronic AOM/DSS model. Since tofacitinib is an oral drug (10 mg twice daily for UC patients) (17), we developed a protocol with tofacitinib in drinking water.

The tofacitinib *in vitro* treatment of CD4^+^ T cells inhibited IFN-γ when added, not only upon activation but also after the first activation and differentiation signals. Therefore, we administered it to mice with DSS-induced colitis either after the first, second, or third DSS cycles or only after the second and third DSS cycles, i.e., starting either early or late during an ongoing intestinal inflammation. Each period with tofacitinib-enriched drinking water lasted 5 days only, but mice were monitored for over 90 days. Interestingly, the treatment with a later onset attained superior protective results in overall clinical IBD scores and in the inhibition of pro-inflammatory cytokines, while the expression levels of anti-inflammatory IL-10 and tissue-healing IL-22 were increased.

## 2 Materials and methods

### 2.1 Mice

*Nfatc1* ^caaA^ (*c.n.Nfatc1*) (22) mice were crossed with distal *Lck* promoter-driven Cre (dLckCre) (23) in order to generate *Nfatc1* ^caaA^.dLckCre mice. Here, the Cre recombinase was expressed under the control of the distal promoter of *Lck*, which is active only in post-thymic T cells. Wild-type (WT) and transgenic mice, male or female, were bred and maintained on a C57BL/6 J background at the Zentrum für Experimentelle Molekulare Medizin (ZEMM), University of Wuerzburg.

### 2.2 CAC induction in mice

C57BL/6 J male/female mice aged 12–16 weeks were chemically induced with DSS after being injected with the procarcinogen AOM (24). Thus, CAC was induced by an intraperitoneal (i.p.) injection of a single dose of AOM (10 mg/1 kg body weight; Sigma) on the first day followed by three cycles of 2% DSS (MP-Biomedicals) in drinking water for 5 days, with an interval of normal drinking water with 0.1% sucrose (see section 2.3) for 5 days. This procedure allowed all mice to survive. After the regimen, the mice were monitored for up to 90 days. Their weights were checked every 5 days and their feces consistencies were scored (from 0 to 8) in accordance with the ethics protocol.

### 2.3 Tofacitinib administration

Tofacitinib citrate (CP-690550) used in the *in vivo* studies was kindly provided by Pfizer Pharma GmbH.

Mice were administered with tofacitinib not with an osmotic pump (25) but orally, while avoiding oral gavages, in accordance with the methods set out in previous studies (12, 26). Tofacitinib is water-soluble and was tolerated in drinking water (**Supplementary Figure 1A, B**; **Supplementary Tables 1, 2**). Treatment was applied by a change of drinking water after a DSS cycle of 5 days, i.e., after the induction of inflammation only. Tofacitinib administration began at different time points but was restricted to the pause of DSS addition. With reference to the drinking pattern of mice, a daily dose of 2.5mg in 6 ml, i.e. a concentration of 0.4 mg/ml of tofacitinib dissolved in 0.1% sucrose water was calculated (**Supplementary Figure 1B**). The untreated mice (colitis group) were given only 0.1% sucrose.

### 2.4 Histologic injury scores

Colon Swiss rolls (27) were fixed in formalin and embedded in paraffin. Sections measuring 2–4 μm were stained with hematoxylin and eosin (H&E) and examined in a blinded manner by a gastrointestinal pathologist. Histologic injury scores (0–6) and the severity of dysplasia were also determined (28, 29).

### 2.5 Immunofluorescence staining of colon

Colon “Swiss rolls” were fixed with 10% buffered formalin for 3 days. Formalin was replaced in the subsequent days to prepare colon Swiss rolls for embedding. The colons were transversely cut, and paraffin blocks were made. The blocks were sectioned (2–4 µm) on slides for immunofluorescence staining. After deparaffinization, slides were cooked under steam (100°C) with antigen retrieval buffer (Tris/EDTA, pH 9) for 45 min, then washed with tris-buffered saline Tween-20 (TBST) and again with tris-buffered saline (TBS) with an interval of 5 min between each wash. The slides were marked with a PAP pen (Dako; N71310-N) and blocked with Antibody Diluent (Dako, S3022) for 1 h in dark humidity chamber. After blocking, primary antibodies CD3 (Dako; A0452 dilution 1:1,000), Ki67 (Invitrogen; 14-5698-82 dilution ratio 1:100), and CD326/epithelial cell adhesion molecule (EpCAM) (Invitrogen; 14-5791-81 dilution 1:400) were used separately on the slides and kept for 1 h in dark and humid chambers. The slides were washed with TBST at intervals of 5 min each. The slides were subjected to secondary antibody AF 488 (Invitrogen; A-21206 dilution 1:400) for CD3 and AF 647 (Invitrogen; A-21247 dilution 1:400) for Ki67, pre-mixed with phosphate-buffered saline (PBS) containing 0.05% Tween20 and kept for 1 h in the dark. The slides were washed with TBST for three time of 5 min interval. Hoechst stain (Sigma; B2261 dilution 1:5,000) and B220 (Biolegend; 103229 dilution 1:100) were applied to the slides and they were then kept for 1 h in dark humidity chamber. After washing with TBST, the slides were mounted with mounting media, Mowiol 4–88 (Roth, #0713). The pictures were captured using an LSM780 confocal microscope (ZEISS, Oberkochen, Germany).

### 2.6 Isolation of lymphocytes from the colon

Colons were harvested from mice, flushed with ice-cold PBS, and cut longitudinally. The colon samples were cut into two to three pieces and incubated with Hanks’ balanced salt solution (HBSS) containing 5 mM ethylenediaminetetraacetic acid (EDTA), 2 mM dithiothreitol (DTT), and 5% fetal bovine serum (FBS) in a shaker at 37°C. The mix containing the colon tissues was vortexed vigorously and passed through a 70 µM cell strainer. The steps were repeated twice and obtained cell suspensions were centrifuged to collect the intraepithelial (IE) cells. The colon tissue was subjected to enzymatic digestion. Tissues were minced followed by a 40 min digestion period at 37°C on a shaking platform in a digestion solution [Roswell Park Memorial Institute medium + 10% FBS containing 1 mM CaCl_2_ and 1 mM MgCl_2_, 0.5mg/mL collagenase D (Roche; 11088882001) and 0.1 mg/mL DNAse I (Roche; 14828300)]. Following the enzymatic digestion, the mix was passed through a cell strainer and mechanically dissociated with the plunger of a syringe, after which the obtained LP-cell suspension was centrifuged. The steps were repeated twice. Finally, a percoll (Cytiva; GE17-0891-01) gradient of 40% (containing the cell suspension) with 80% was used to separate the lymphocytes in the buffy coat from the isolated IE and LP cells.

### 2.7 Flow cytometry

Cells were washed once in flow assisted cell sorting (FACS) buffer [PBS containing 0.1% bovine serum albumin (BSA)] before blocking with anti-FcγRII/FcγRIII (2.4G2, BD Pharmingen). Staining of surface molecules (all Biolegend antibodies) was performed at room temperature using CD4 (RM4-5), CD8α (53-6.7), and CD25 (PC61), TNFRII (TR75-89), and intracellular Foxp3 (FJK-16s, eBioscience). T-bet (4B10, Biolegend) staining was performed using the Foxp3 staining kit (eBioscience) in accordance with the manufacturer’s instructions. The antibodies (all Biolegend) used for intracellular cytokine staining were IFN-γ (XMG1.2); TNF-α (MP6-XT22); IL-2 (JES6–5H4); IL-17A (TC11–18H10.1), and IL-10 (JES5–16E3) using IC Fixation Buffer kit (eBioscience). Cytokine detection was performed after a 6 h *in vitro* restimulation with 12-O-tetradecanoylphorbol-13-acetate (TPA; 10 ng/mL, Sigma) plus ionomycin (5 nM, Merck Biosciences) in the presence of GolgiStop and GolgiPlug (both BD Pharmingen). Viable cells were detected with the Zombie Aqua™ Fixable Viability Kit (Biolegend). Data were acquired on a FACSCanto II (BD Biosciences) flow cytometer and analyzed with FlowJo software (Tree Star).

### 2.8 Cytokine analysis of murine cell supernatants and sera

To determine various cytokines in supernatants from differentiated clusters of differentiation 4 (CD4^+^) T cells, a bead-based immunoassay (LEGENDplex™) was performed, in accordance with instructions in the manufacturer’s manual. Samples were measured and analyzed using flow cytometry. Data analysis was performed in accordance with the instructions provided by the Analysis Software LegendPlex 7.0.

To determine the concentration of circulating cytokines, sera were collected from the mice using Micro Serum-Gel, 1,1 mL (Sarstedt: 41.1500.005) and by centrifuging at 2,500G for 5 min. The collected serum was stored. The recommended protocol for LEGENDplex Mouse Th panel-12 plex V03 (Cat. 741043) was used to analyze and estimate the concentration of cytokines. Legendplex Qognit software was used to determine the concentration of cytokines.

### 2.9 *In vitro* differentiation of murine CD4^+^ T cells

Isolated CD4^+^ T cells (immunomagnetic negative selection, stemcell™ technologies) were differentiated in Iscove’s modified Dulbecco’s medium (IMDM) by the addition of cytokine mixtures during stimulation. Prior to cell sowing, the culture plate was coated with anti-hamster immunoglobin G (IgG) (1:200 in PBS w/o divalent salts) for 2 h at 37°C. The antibody solution was removed, and the plate was washed two times with PBS (without divalent salts). CD4^+^ T cells were seeded at a concentration of 1 × 10^6^/mL. Anti-CD3ϵ (0.5 µg/mL), anti-CD28 (2 µg/mL), binding to anti-hamster IgG, and cytokines (all Peprotech) for Th1 or Th17 differentiation were added: Th1 - human interleukin-2 (hIL-2) (10 ng/mL), mouse interleukin-12 (mIL-12) (10 ng/mL), and human interferon γ (hIFN-γ) (50 ng/mL); Th17 - human-transforming growth factor beta type 2 (hTGF-β) (2.5 ng/mL), mIL-6 (50 ng/mL), hIL-21 (62.5 ng/mL), anti-mIFN-γ (5 µg/mL), and anti-mIL-4 (5 µg/mL). After 2 days, the stimulus was removed and the cells transferred to new plates. After a further 3 days, the cells and supernatants were harvested.

### 2.10 Statistical analysis

All figures were prepared using Corel Draw software and the graphs were evaluated using GraphPad Prism 8 software. Different groups were compared by either an unpaired Student’s *t*-test or a Mann–Whitney test using GraphPad Prism 8 software. Differences for *p*-values of > 0.05 were considered not significant, but *p*-values of ≤ 0.05 were considered significant and indicated in figures as *p* ≤ 0.05 (*), *p* ≤ 0.01 (**), and *p* ≤ 0.001 (***). Replicates were specified in the figures as individual mice or experiments.

## 3 Results

### 3.1 Tofacitinib potently inhibited IFN-γ production of CD4^+^ T cells *in vitro*

In IBD, CD4^+^ Th1 and Th17 cells are known to be induced, resulting in a pro-inflammatory cytokine expression pattern. The cytokine expression of T cells is primarily determined by cytokine milieu *via* signaling of the respective cytokine receptors. Thus, the impact of JAK/signal transducer and activator of transcription (STAT) inhibition on T-helper type 1 (Th1) and Th17 cell differentiation was determined before starting with the murine IBD model. We skewed CD4^+^ T cells toward Th1 and Th17 and analyzed their cytokine secretion in the time-restricted presence of tofacitinib. More precisely, tofacitinib was constantly present for only the first 2 days of initial stimulation and cytokine addition, or only for the last 3 days when taken off the stimulus.

**Figure 1:**
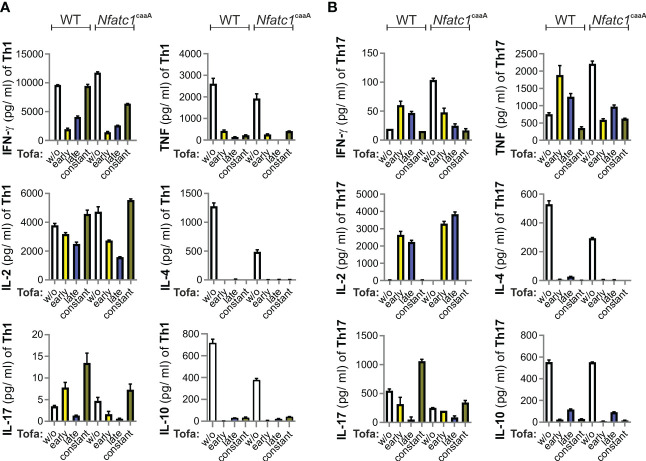
Pulses of tofacitinib during Th1 cell differentiation *in vitro* limit IFN-γ, but not IL-2 production. CD4^+^ T cells from WT and *Nfatc1* ^caaA^ dLckCre mice were stimulated with α-CD3 and α-CD28 and skewed toward Th1 **(A)** or Th17 **(B)** by Th1/Th17-promoting cytokines. At the indicated time intervals, 0.1 nM tofacitinib was present; early (days 1 and 2), late (days 3–5), and constant (days 1–5). The number of cytokines was evaluated in culture supernatants. Two independent experiments were carried out, each in triplicate. IFN‐γ, interferon gamma; IL-2, interleukin-2; Th1, T-helper type 1; WT, wild type.

Tofacitinib potently inhibited the IFN-γ secretion of Th1 cells when given for the first 2 days or the last 3 days (**Figure 1A**). In Th1 cells, TNF secretion was blocked completely. Th17 cells responded to tofacitinib pulses (both during days 1–2 and 3–5) with a tendency to lower IL-17A, a slight upregulation of IFN-γ, and high amounts of IL-2 and TNF (**Figure 1B**). In addition, IL-4 and IL-10 expression was lost under all tofacitinib regimes.

We compared the T cells of WT mice with those of genetically engineered mice (*Nfatc1* ^caaA^. dLckCre), which constitutively express an active version of the short isoform of nuclear factor of activated T cells 1 (NFATc1), caNFATc1/αA (30), in peripheral T cells. The expression of caNFATc1/αA resembles chronic inflammation (31–34), i.e., the pathophysiological state of patients. Nevertheless, tofacitinib additions revealed very similar patterns in WT and caNFATc1/αA^+^ T cells (**Figures 1A, B**). Taken together, the most pronounced direct effects of tofacitinib on pro-inflammatory CD4^+^ T cells were the inhibitions of IFN-γ, IL-4, and IL-10. In contrast, TNF expression was context-dependent and IL-2 could even be enriched by short intervals of tofacitinib application.

### 3.2 Late administration of tofacitinib ameliorated chronic DSS colitis

**Figure 2:**
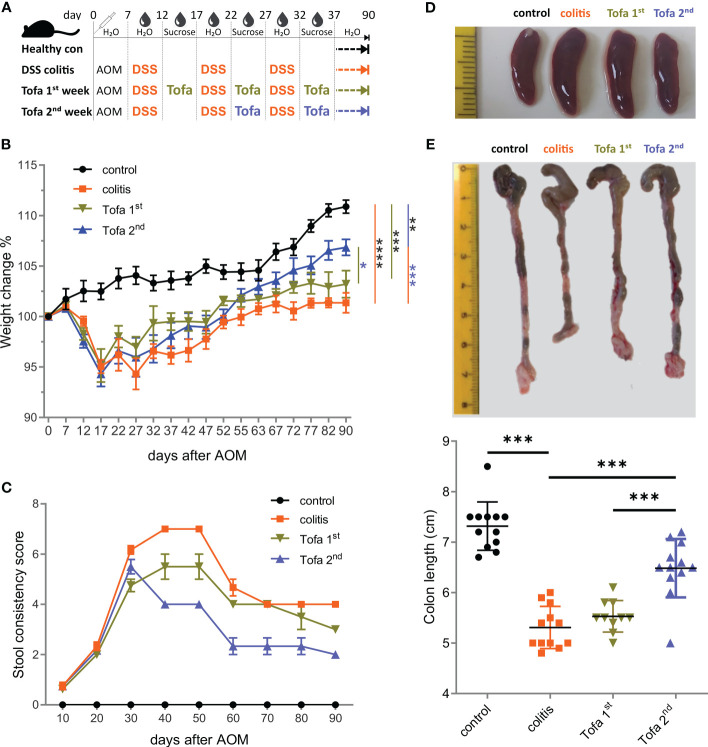
Early onset tofacitinib treatment is less beneficial for IBD-induced mice than later-onset treatment. **(A)** C57BL/6 mice aged 12–16 weeks were subjected to three treatment cycles with 2% DSS following AOM and observed for 90 days. Tofacitinib was given intermittently with DSS, starting either after the first or after the second cycle. **(B)** The weights of all mice upon the start of the experiment and on indicated days. **(C)** The score of stool consistency on indicated days. **(D)** Representative images of spleens from each group. **(E)** Colons straightened, but not stretched and aligned at the ileocecal junction. The colon length from the ileocecal junction to the distal end of the rectum was measured; mean ± SD, unpaired Student’s *t*-test (*p ≤ 0.05, **p ≤ 0.01, ***p ≤ 0.001, and ****p ≤ 0.0001). Data are representative of three independent experiments; at least *n* = 3 in each group. AOM, azoxymethane; DSS, dextran sulfate sodium; IBD, inflammatory bowel disease.

Since tofacitinib was shown to reduce the IFN-γ levels of *in vitro* differentiated Th1 cells (**Figure 1A**), we were confident in administering it *in vivo* during ongoing IBD, either starting after the first (Tofa 1st) or second (Tofa 2nd) cycle of DSS induction ending up with two or three 5-day intervals of tofacitinib-enriched drinking water (**Figure 2A**). Indeed, this JAKinib was able to reduce the IBD symptoms measured as clinical scores (**Supplementary Figure 2A**), i.e., weight change (**Figure 2B**) and stool consistency (**Figure 2C**). We also observed that in treated mice, splenomegalies were pronounced, and colons were thickened and shortened to a lesser extent, which is an indication of disease protection (**Figures 2D, E**). Remarkably, providing tofacitinib at a later stage, i.e., only after the second and third DSS cycles, resulted in the mice being healed to a greater extent than that of the earlier start after the first DSS cycle and the sum of three application periods.

### 3.3 More limited administration of tofacitinib-protected colonic tissue

**Figure 3:**
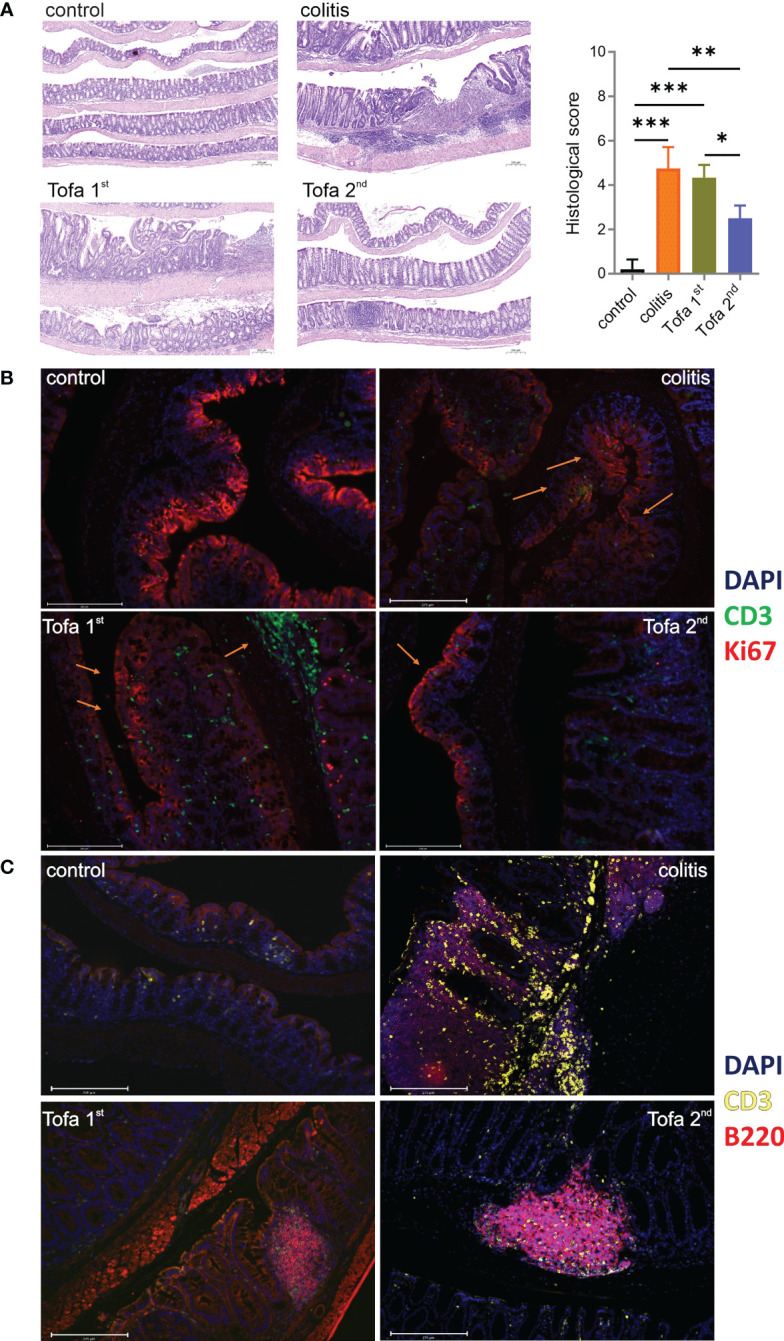
Tofacitinib protects from intestinal inflammation and destruction when given later during the course of developing colitis. Colons were opened longitudinally and rolled with the mucosa outwards into a Swiss roll, fixed, and embedded in paraffin. **(A)** Sections 2–4 µm in length were stained with H&E (blue indicates nuclei and purple indicates cytoplasm/matrix) and observed at × 50 magnification to determine their histologic scores; scale bar 200 µm; mean ± SD, unpaired Student’s *t*-test (*p ≤ 0.05, **p ≤ 0.01, ***p ≤ 0.001. Data are representative of three independent experiments; at least *n* = 3 in each group. **(B)** IF staining with DAPI (blue; nuclei), anti-CD3 (green; T cells), and Ki67 (red; proliferating cells) at × 20 magnification. **(C)** IF staining with DAPI, anti-CD3 (yellow), and anti-B220 (red; B cells) at × 20 magnification. The scale bar shows 200 µm. DAPI, 4′,6-diamidino-2-phenylindole; H&E, hematoxylin and eosin; IF, immunofluorescence.

Ninety days after the initial AOM injections, Swiss roll colons were prepared (27) from both healthy controls and DSS-induced IBD mice, either untreated or given three (Tofa 1st) *vs.* only two (Tofa 2nd) cycles of tofacitinib in drinking water (**Figure 2A**). The H&E staining of colons demonstrated that mice that received tofacitinib early and more often suffered from colon tissue destruction in almost the same manner as untreated colitis-induced mice (**Figure 3A**; **Supplementary Figure 3A**). On the other hand, administration at a later stage and to a lesser extent fully preserved or restored the intactness of intestinal tissues.

In contrast to that observed in disease-free control mice, the expression of proliferation-associated Ki67 was not limited to the crypts in colons of colitis-induced mice but was apparent throughout the intestinal tissue as increasing levels of Ki67 expression in the apical epithelium are suggestive of dysplasia (**Figure 3B**). Whereas the early administration of tofacitinib led to substantially less Ki67 expression, indicating less inflamed and destructed epithelia, infiltration of CD3^+^ T cells was high. Only Tofa 2nd ensured the healthy restriction of Ki67 expression in crypts. In comparison to healthy mice, the infiltration of T lymphocytes between the epithelial linings was massive and frequently observed in colitis-induced mice (**Figures 3B, C**). The colons of tofacitinib-treated mice showed less abundant T-cell infiltrations, in addition to the obvious dominance of B cells over T cells. Here, early-onset tofacitinib treatment resulted in a mixture of T and B cells being spread over the whole tissue, whereas later treatment revealed even fewer T cells and B cells confining themselves to B-cell aggregates, probably ectopic lymphoid follicles.

### 3.4 Late administration of tofacitinib-supported TNFRII^+^ Tregs

**Figure 4:**
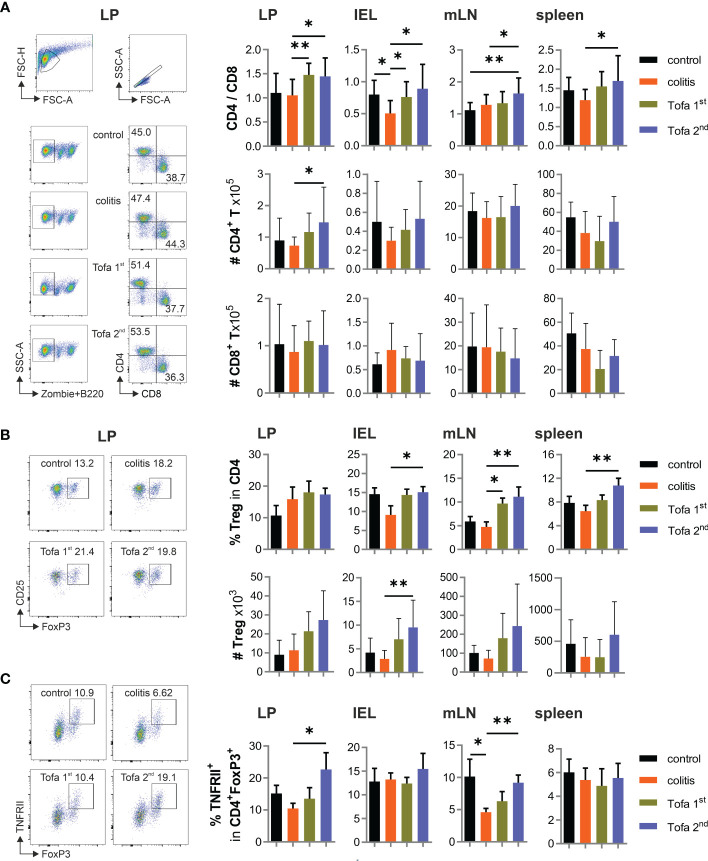
Tofa 2nd group shows higher protective capacity with more active Tregs. **(A)** The ratio of the percentage of CD4/CD8 and their absolute numbers. Representative gating strategy of LP and graphs of LP, IELs, mLNs, and spleen. **(B)** Representative dot plot with LP cells and the relative and absolute numbers of Tregs in different organs. **(C)** Frequency of TNFRII^+^ Tregs; mean ± SD, unpaired Student’s *t*-test (*p ≤ 0.05, **p ≤ 0.01. Data are representative of three independent experiments. IELs, intraepithelial lymphocytes; LP, lamina propria; mLNs, mesenteric lymph nodes; Tregs, T-regulatory cells.

We also collected T cells from colon LP, among intraepithelial lymphocytes (IELs), mLNs, and spleens from mice of all groups after 90 days. The ratio of CD4^+^ to CD8^+^ T cells increased due to tofacitinib treatment, we also observed that the frequency of CD4^+^ regulatory T cells (Tregs) within CD4^+^ T-cell populations was elevated (**Figures 4A, B**). The latter was especially true when tofacitinib therapy was started later, i.e., after the second DSS cycle. Since TNF production is decisively upregulated in IBD and could not be well suppressed by tofacitinib in Th17 cell cultures (**Figure 1B**), we questioned the role of TNF in affecting Tregs and determined the level of beneficial TNFRII expression on Tregs. Indeed, while IBD induction tended to reduce the numbers of TNFRII^+^ Tregs, later-onset tofacitinib treatment triggered their recovery in intestinal tissues (**Figure 4C**).

### 3.5 Late administration of Tofacitinib to colitis mice reduced the abundance of IFN-γ^+^ T cells and conversely enriched IL-10^+^ T cells

**Figure 5:**
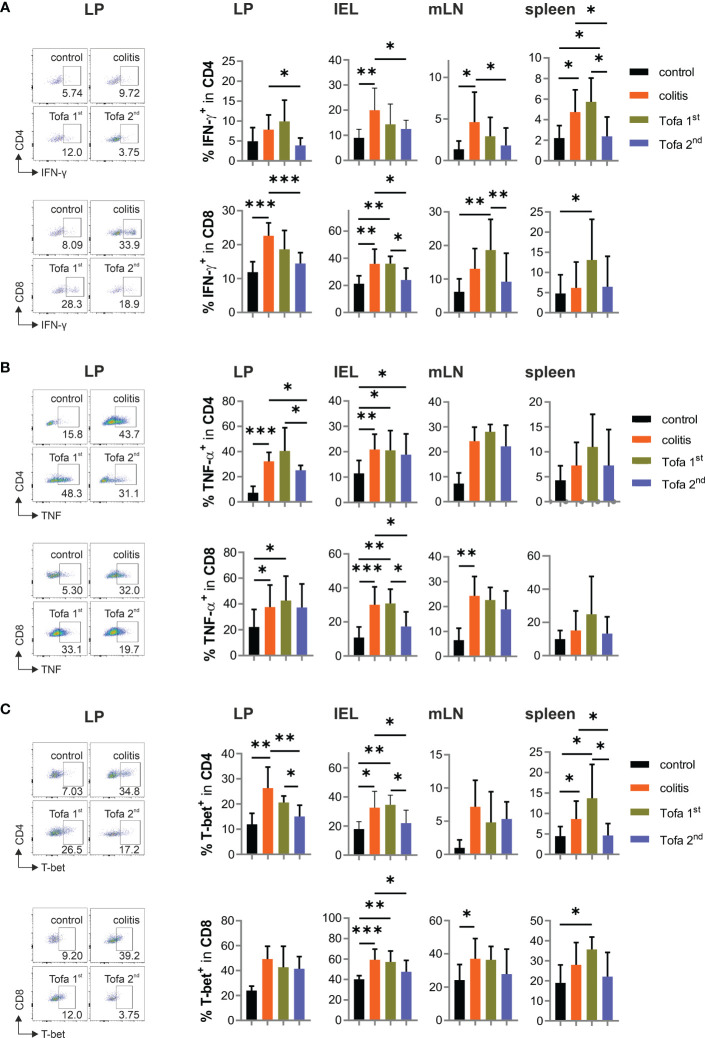
Pro-inflammatory cytokines are significantly reduced in Tofa 2nd group compared to the colitis group. Representative dot plots of LP and statistical analysis of relative numbers from LP, IELs, mLNs, and spleen of CD4^+^ and CD8^+^ T cells expressing IFN-γ **(A)**, TNF-α **(B)**, and T-bet **(C)**; mean ± SD, unpaired Student’s *t*-test (*p ≤ 0.05, **p ≤ 0.01, ***p ≤ 0.001. Data are representative of three independent experiments. IELs, intraepithelial lymphocytes; IFN‐γ, interferon gamma; LP, lamina propria; mLNs, mesenteric lymph nodes; TNF-α, tumor necrosis factor-alpha.

In addition, we assessed the cytokine expression of CD4^+^ and CD8^+^ T cells in the aforementioned tissues in all groups of mice. For this, T cells were shortly re-stimulated by TPA/ionomycin, which then indicated their *in vivo* cytokine expression potential. As predicted, levels of IFN-γ-expressing T cells increased due to chronic IBD; however, these could be reliably reverted by late-onset tofacitinib treatment in drinking water (**Figure 5A**). In parallel, the frequency of TNF^+^ T cells increased due to DSS induction, which could only be reduced in CD4^+^ T cells of the LP and CD8^+^ T cells among IELs by the late-stage provision of tofacitinib (**Figure 5B**). In contrast, starting medication after the first DSS cycle was inefficient, and in some tissues, it even triggered higher levels of IFN-γ^+^ T cells than in those of colitis mice. Corresponding with IFN-γ expression, the frequency of T-bet^+^ T cells was elevated during DSS colitis, which—especially in CD4^+^ T cells—could be limited by the late administration of tofacitinib medication (**Figure 5C**). A reduced expression of the key transcription factor for Th1 cells demonstrated a lasting change in T-cell differentiation after two pulses of tofacitinib.

**Figure 6:**
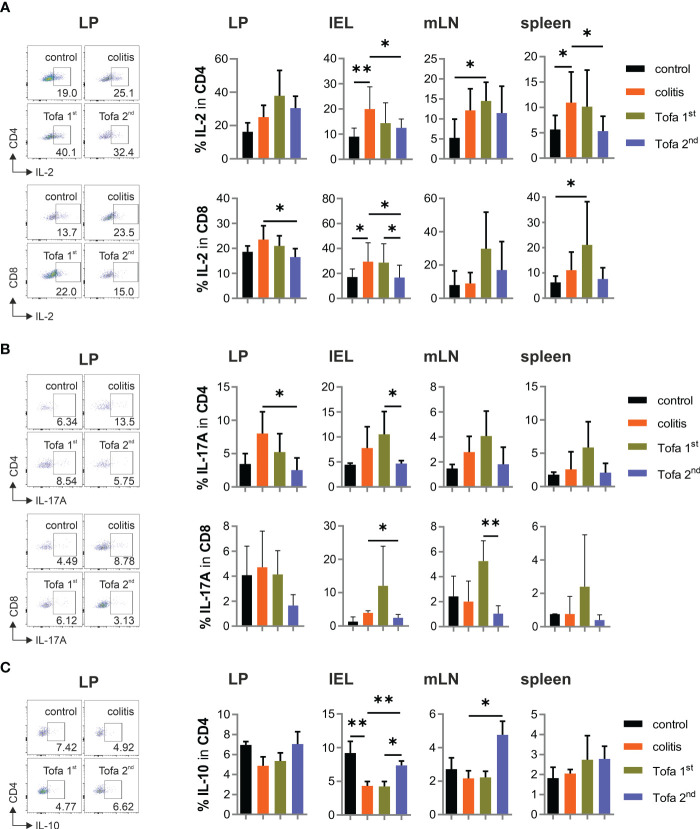
While the frequency of IL-17^+^ T cells decreases, IL-10^+^ CD4^+^ T cells increase in the Tofa 2nd group. Representative dot plots of LP and statistical analysis of relative numbers from LP, IELs, mLNs, and spleen of CD4^+^ and CD8^+^ T cells expressing IL-2 **(A)** or IL-17 A **(B)** and CD4^+^ T cells expressing IL-10 **(C)**; mean ± SD, unpaired Student’s *t*-test (*p ≤ 0.05, **p ≤ 0.01. Data are representative of three independent experiments. IELs, intraepithelial lymphocytes; LP, lamina propria; mLNs, mesenteric lymph nodes.

Although IL-2 expression was not directly repressed by tofacitinib *in vitro*, the heightened frequency of IL-2^+^ T cells in colitis-diseased mice was partly controlled by later, but less so by earlier, tofacitinib administration (**Figure 6A**). The same was true for the frequency of IL-17A^+^ T cells (**Figure 6B**). In contrast to the direct *in vitro* suppression of IL-10 expression by tofacitinib, however, the number of IL-10^+^ CD4^+^ T cells in colitis mice on day 90 could not only be reverted by the late administration of tofacitinib but also sometimes even exceeded the levels observed in control mice (**Figure 6C**).

In sum, providing tofacitinib in drinking water after the second and third DSS cycles protected mice from high expressions of pro-inflammatory cytokines—especially IFN-γ—and allowed the enrichment of T cells with anti-inflammatory IL-10.

### 3.6 Late medication with tofacitinib allowed IL-22 expression but prevented EpCAM^+^ CAC

**Figure 7:**
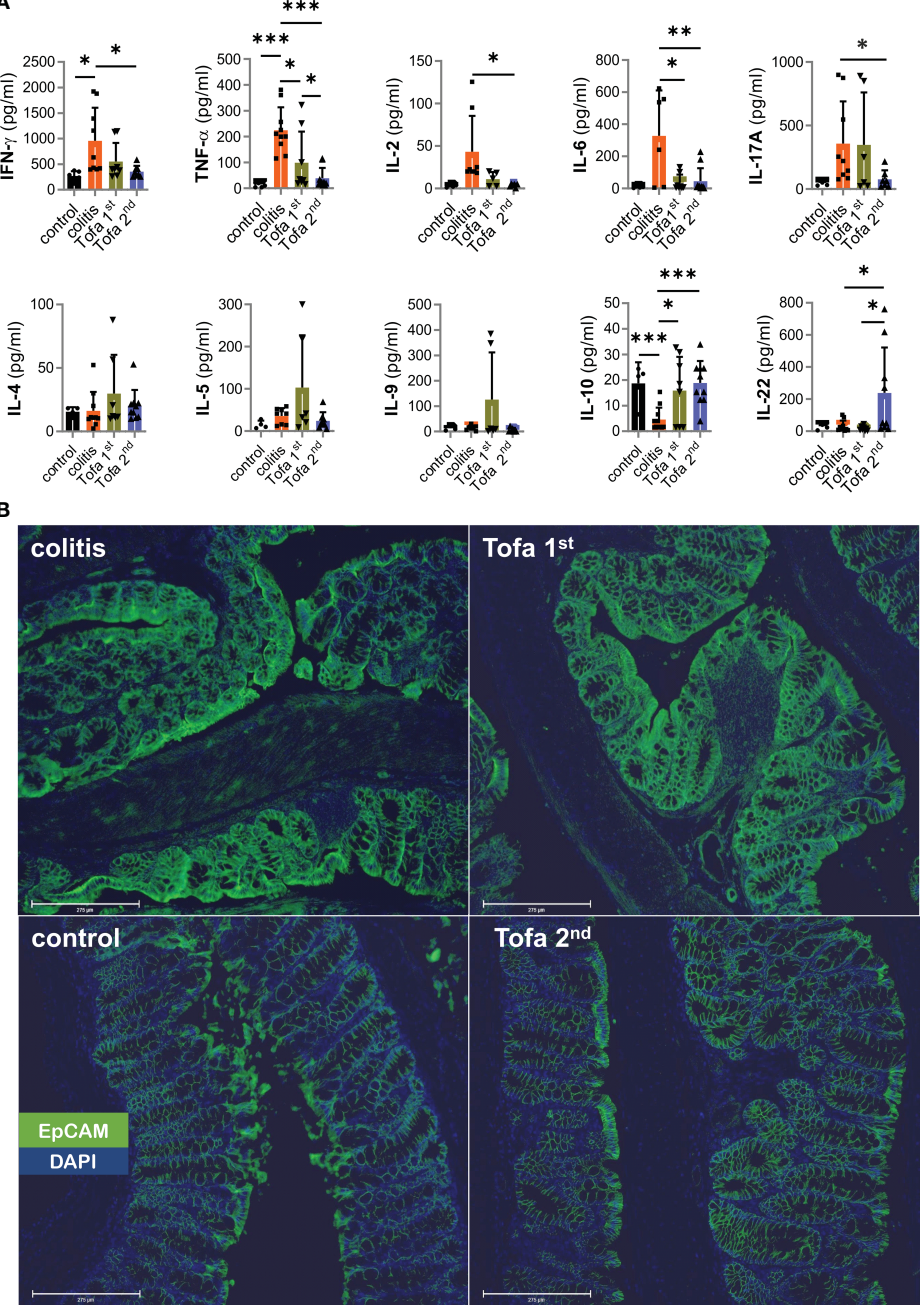
Systemic upregulation of IL-22 correlates with the prevention of EpCAM overexpression. **(A)** Quantitative measurement of cytokines from the sera collected from mice; Mean ± SD, unpaired Student’s *t*-test (*p ≤ 0.05, **p ≤ 0.01, ***p ≤ 0.001. Data are representative of three independent experiments; **(B)** Representative example of the qualitative expression of EpCAM (green) and DAPI (blue) in all four groups. Scale bars equal 275 µm at × 20 magnification. DAPI, 4′,6-diamidino-2-phenylindole; EpCAM, epithelial cell adhesion molecule.

Pro-inflammatory T cells are the main drivers of IBD. Nevertheless, many other cells also secrete cytokines. In addition, secreted cytokines can either diminish or accumulate over time. Therefore, we assessed the serum level of cytokines at the end of our colitis experiments (**Figure 7A**), and found that IFN-γ, TNF, IL-2, IL-6, and IL-17 levels were all much higher in DSS-induced colitis mice than in control mice. Interestingly, while both tofacitinib treatment regimens clearly led to reduced levels of cytokines in sera, those for the later onset regimen were lowest. In contrast, levels of Th2 cytokines—although low in general—were highest after earlier-onset tofacitinib treatment.

Reflecting the expression potential in T cells *ex vivo*, IL-10 serum levels contracted in DSS-colitis mice. In contrast to direct *in vitro* data (**Figures 1A, B**) but in line with *ex vivo* T-cell data (**Figure 6C**), IL-10 serum levels recovered with tofacitinib treatment, even reaching those of control mice with the later-onset treatment (**Figure 7A**).

We included IL-22 measurements since IL-22—produced by several populations of immune cells at sites of inflammation—has tissue-healing properties (35). This regimen greatly increased IL-22 levels, which is encouraging for the later onset protocol of tofacitinib administration and is consistent with the mucosal healing that was seen (**Figures 7A**, **3A–C**).

The intention of the protocol with an AOM injection prior to colitis induction by DSS was to enforce CAC development. Levels of the critical tumor-promoting TNF and IL-6 sera were low, but mucosal-healing IL-22 also supports tumor formation (35). We scanned colons and found hardly any pseudopolyps, which resemble adenoma or cancer macroscopically, after later onset-tofacitinib administration (**Supplementary Figure 3B**). Therefore, we stained for EpCAM, CD326, identifiable by its high expression on rapidly proliferating carcinoma of epithelial origin (36). Obviously, AOM/DSS-colitis mice that had not received, but also those that had received, early-onset tofacitinib treatment showed high EpCAM expression; whereas those that had received merely the late-onset tofacitinib treatment, i.e., after the second DSS cycle, showed normalized EpCAM expression levels compared with those of control mice (**Figure 7B**). Still, no EpCAM^+^ carcinoma cells beyond lamina muscularis mucosae could be clearly detected, and thus increased EpCAM expression in colitis and Tofa 1st colons indicated highly inflamed tissue with T cells and antigen-presenting cells (37).

Altogether, the JAKinib tofacitinib, when provided not too early, is very efficient in limiting clinical signs of DSS-induced colitis and the expression of pro-inflammatory cytokines, while supporting Treg cells, IL-10 and IL-22 expression.

## 4 Discussion

Tofacitinib, which is a pan-JAKinib, was effective in restoring intestinal intactness in a chronic IBD model, induced by three cycles of DSS following injection of the procarcinogen AOM. In addition, the pseudopolyp appearance and EpCAM expression were normalized in intestinal epithelial cells, also demonstrating the reduced likelihood of CAC development. When the DSS cycle-intermitting provision of tofacitinib was started after the first DSS administration, only limited positive effects such as normalized stool consistencies or colon length were achieved, and the production of proinflammatory cytokines could not be restricted. EpCAM expression appeared to be as severe as in the AOM/DSS colitis mice, for example, in line with heightened IL-9 levels (38). However, tofacitinib medication after the second and third DSS cycles entailed betterment on all levels.

In addition to T-cell receptor (TCR) and costimulatory signals, T cells receive the so-called third signal, which is delivered by the cytokines secreted by the primarily activated innate immune cells. Signals 1–3 can be individually addressed by specific drugs (39). Most cytokines induce JAK/STAT signaling *via* their respective cytokine receptors. The particular network of JAKs/STATs then dictates the differentiation toward CD4^+^ or CD8^+^ T-cell subsets, which are themselves characterized by an individual set of secreted cytokines. The influence of the innate immune system and the subsequent reinforcement by autocrine cytokines is already so complex that the *in vivo* effects of JAKinibs like tofacitinib is not easily predicted.

Innate-derived, immune response-initiating cytokine provisions are routinely mimicked by exogenous cytokine additions to T-cell cultures. During Th1 differentiation, tofacitinib suppressed not just IFN-γ, as reported before (12) but also TNF. When we skewed toward Th17, however, the leading cytokine IL-17 was negatively affected, but TNF expression was less so. IFN-γ expression depends on the IL-12-activated STAT4 and is reinforced by the IFN-γ-activated STAT1. IL-17 is also influenced by the STAT3-activating cytokines IL-6 and IL-21. TNF, on the other hand, is an immediate response gene to TCR plus co-stimulation (40, 41). As a result, the failure of tofacitinib inhibition meets expectations. The corepression with IFN-y in Th1 cells could be the result of a general inhibition of differentiation. The picture seemed similarly complex *in vivo*, in that while most T cells could still express TNF *ex vivo* when re-stimulated by TPA/ionomycin, the sera of tofacitinib-treated mice revealed reduced levels of TNF. Hence, 7 weeks after tofacitinib treatment was stopped, although T cells were capable of producing TNF, there were no mechanisms inducing this, since the colitis was mostly resolved.

Another important lymphokine, which has rapid primary expression and is not influenced by JAK/STAT activation, is IL-2 (42). In line with this, the secretion of IL-2 by Th1 cells was rather unchanged by the addition of tofacitinib. In Th17 cell cultures, the pulses of tofacitinib even led to dramatically increased levels of secreted IL-2 in culture supernatants, most likely because of dominating TCR and co-stimulatory signals in the context of failing Th17 differentiation. This is reassuring for IBD treatment since the total loss of IL-2 causes severe bowel inflammation (43).

In contrast to its induction, however, IL-2 itself signals *via* STAT5 (44). IL-2 supports Th1 and Th2 cells to become effector T cells, while it counteracts Th17 and T_FH_ differentiation (44, 45). Most importantly, however, IL-2 is decisive for Tregs being required for their differentiation and homeostasis (46), which is demonstrated by substantially reduced Treg populations in mice lacking either STAT5 or JAK3 (47). Accordingly, interference at too early a stage, for example with a JAKinib, must destabilize those Treg populations. Indeed, delayed tofacitinib and, especially, late-onset treatment led to heightened frequencies of Foxp3^+^CD25^+^ Tregs that were still evident 7 weeks after the last tofacitinib cycle. At that time point, conventional T cells demonstrated a reduced potential to express IL-2, and IL-2 serum levels were low in mice that had received tofacitinib. In contrast, we have demonstrated before that a time-restricted increase in IL-2 during the onset of an autoimmune disease or alloreactive response is beneficial for the overall outcome of such diseases (48). In both scenarios, the increase in Treg levels was key.

When the disease progresses, Tregs further differentiate into effector Tregs, in which homeostasis no longer depends on IL-2/STAT5 signals (49). One supportive feature could be the identified upregulation of TNFRII, which is induced upon T-cell receptor activation on CD25^+^Foxp3^+^ Tregs (50) and is subsequently one of their key regulators under inflammatory conditions (51). Hence, the later onset of tofacitinib medication gives IL-2 enough time to expand Tregs, which in turn upregulate effector molecules to be instrumental in controlling the pro-inflammatory immune response, and also facilitates tissue protection and healing.

However, the provision of Tregs would be wholly ineffective if, as has previously been found in IBD patients, conventional T cells are resistant to Treg-mediated suppression (52). Therefore, tofacitinib can play a supportive role by blocking JAK/STAT-dependent pro-inflammatory cytokines by facilitating the expression of anti-inflammatory cytokines. Reassuringly, IL-10 serum levels and their expressions in T cells were restored in colitis mice when they underwent late onset tofacitinib treatment although tofacitinib had been shown to completely abolish the expression of this STAT3-dependent cytokine in culture. IL-10 is secreted by Tregs, although not exclusively, but is differently regulated in different cell types (53). In intestinal thymic-derived and peripherally induced Tregs, TGF-β mediates IL-10 production, which could also be true for differentiated Th1 cells (54, 55). The importance of IL-10 expression for the intactness of the gut became obvious in mice deficient in IL-10, which showed spontaneous gut inflammation (56, 57), and by treatment with IL-10, which decreases the level of pro-inflammatory cytokines like TNF and IL-6 (58).

Another IL-10 family member that has been reported to limit IBD is IL-22 (59, 60). IL-22 is preferentially generated by various immune cells at mucosal sites, induced by different pathways and transcription factors comprising IL-23 receptor-activated STAT3 (35). It plays a critical role in maintaining the epithelial integrity of the intestinal tract and also in the reversal of intestinal epithelial cell destruction occurring in IBD. Like IL-10, IL-22 sends signals *via* STAT3 (and STAT1 and 4) activation, which is only possible after the termination of treatment with tofacitinib. IL-22 surely is involved in CAC development (9), but at least in our model was not sufficient to support CACs as heightened serum levels of IL-22 were reciprocal to enforced EpCAM expression.

Others have claimed that early administration of tofacitinib is not effective, but experimented with a possible preventive effect during acute (1 cycle) DSS-induced colitis (26). When they applied tofacitinib right after DSS, they observed some improvement, but being minor compared to the results we could achieve with late treatment onset in the chronic DSS-induced colitis. Possibly, those positive effects in the acute model (26) were similar to the ones we achieved with an early-onset treatment, i.e., that beginning right after the first DSS cycle. The selected chronic model here reflects relapsing IBD in humans better, involves the adaptive immune system, and allows—by the injection of AOM—the possible study of IBD-associated neoplasia (61). Obviously, as patients are unlikely to receive preventive treatment, they will seek medication for long-lasting and relapsing–remitting IBD, for which we can predict that tofacitinib will be effective. Whereas treatment-naive immediate responses in IBD include IL-2-propagated Tregs, resolving anti-inflammatory and tissue healing actions by IL-10 and IL-22 requires JAK/STAT signaling. Thus, the outcome of our study is that confined treatment cycles with tofacitinib might lead to longer-lasting results.

## Data availability statement

The original contributions presented in the study are included in the article/**Supplementary Material**. Further inquiries can be directed to the corresponding author.

## Ethics statement

The animal study was reviewed and approved by Regierung von Unterfranken (government of Lower Franconia) (55.2.2-2532-2-656).

## Author contributions

RS, LS, CC, NH, GK, and SR-H performed research and analyzed and discussed the data; RS took part in writing the manuscript; AR offered resources and provided financial support; JW hypothesized the oral application of tofacitinib *via* drinking water; FB-S conceptualized the research goals, acquired funding, designed research, discussed the data, and wrote the manuscript. All authors contributed to the article and approved the submitted version.

## References

[1] Schlegel N Boerner K Waschke J. Targeting desmosomal adhesion and signalling for intestinal barrier stabilization in inflammatory bowel diseases-lessons from experimental models and patients. Acta Physiol (Oxf) (2021) 231(1):e13492. doi: 10.1111/apha.13492 32419327

[2] Torres J Mehandru S Colombel JF Peyrin-Biroulet L. Crohn's disease. Lancet (2017) 389(10080):1741–55. doi: 10.1016/S0140-6736(16)31711-1 27914655

[3] Maloy KJ Powrie F. Intestinal homeostasis and its breakdown in inflammatory bowel disease. Nature (2011) 474(7351):298–306. doi: 10.1038/nature10208 21677746

[4] Fava F Danese S. Intestinal microbiota in inflammatory bowel disease: friend of foe? World J Gastroenterol (2011) 17(5):557–66. doi: 10.3748/wjg.v17.i5.557 PMC3040327 21350704

[5] Baumgart DC Carding SR. Inflammatory bowel disease: cause and immunobiology. Lancet (2007) 369(9573):1627–40. doi: 10.1016/S0140-6736(07)60750-8 17499605

[6] Neurath MF. Cytokines in inflammatory bowel disease. Nat Rev Immunol (2014) 14(5):329–42. doi: 10.1038/nri3661 24751956

[7] Abraham C Cho JH. Inflammatory bowel disease. N Engl J Med (2009) 361(21):2066–78. doi: 10.1056/NEJMra0804647 PMC3491806 19923578

[8] Yao D Dong M Dai C Wu S. Inflammation and inflammatory cytokine contribute to the initiation and development of ulcerative colitis and its associated cancer. Inflammation Bowel Dis (2019) 25(10):1595–602. doi: 10.1093/ibd/izz149 31287863

[9] Francescone R Hou V Grivennikov SI. Cytokines, IBD, and colitis-associated cancer. Inflammation Bowel Dis (2015) 21(2):409–18. doi: 10.1097/MIB.0000000000000236 PMC4481731 25563695

[10] Danese S Colombel JF Peyrin-Biroulet L Rutgeerts P Reinisch W. Review article: the role of anti-TNF in the management of ulcerative colitis – past, present and future. Aliment Pharmacol Ther (2013) 37(9):855–66. doi: 10.1111/apt.12284 23489068

[11] van Dullemen HM van Deventer SJ Hommes DW Bijl HA Jansen J Tytgat GN. Treatment of crohn's disease with anti-tumor necrosis factor chimeric monoclonal antibody (cA2). Gastroenterology (1995) 109(1):129–35. doi: 10.1016/0016-5085(95)90277-5 7797011

[12] Ghoreschi K Jesson MI Li X Lee JL Ghosh S Alsup JW. Modulation of innate and adaptive immune responses by tofacitinib (CP-690,550). J Immunol (2011) 186(7):4234–43. doi: 10.4049/jimmunol.1003668 PMC3108067 21383241

[13] Ghoreschi K Laurence A O'Shea JJ. Janus kinases in immune cell signaling. Immunol Rev (2009) 228(1):273–87. doi: 10.1111/j.1600-065X.2008.00754.x PMC2782696 19290934

[14] O'Shea JJ Pesu M Borie DC Changelian PS. A new modality for immunosuppression: targeting the JAK/STAT pathway. Nat Rev Drug Discovery (2004) 3(7):555–64. doi: 10.1038/nrd1441 15232577

[15] Danese S Grisham M Hodge J Telliez JB. JAK inhibition using tofacitinib for inflammatory bowel disease treatment: a hub for multiple inflammatory cytokines. Am J Physiol Gastrointest Liver Physiol (2016) 310(3):G155–62. doi: 10.1152/ajpgi.00311.2015 PMC4971816 26608188

[16] Meyer DM Jesson MI Li X Elrick MM Funckes-Shippy CL Warner JD. Anti-inflammatory activity and neutrophil reductions mediated by the JAK1/JAK3 inhibitor, CP-690,550, in rat adjuvant-induced arthritis. J Inflammation (Lond) (2010) 7:41. doi: 10.1186/1476-9255-7-41 PMC2928212 20701804

[17] Sandborn WJ Su C Sands BE D'Haens GR Vermeire S Schreiber S. Tofacitinib as induction and maintenance therapy for ulcerative colitis. N Engl J Med (2017) 376(18):1723–36. doi: 10.1056/NEJMoa1606910 28467869

[18] Sattler L Hanauer SB Malter L. Immunomodulatory agents for treatment of patients with inflammatory bowel disease (Review safety of anti-TNF, anti-integrin, anti IL-12/23, JAK inhibition, sphingosine 1-phosphate receptor modulator, azathioprine / 6-MP and methotrexate). Curr Gastroenterol Rep (2021) 23(12):30. doi: 10.1007/s11894-021-00829-y 34913108

[19] Okayasu I Hatakeyama S Yamada M Ohkusa T Inagaki Y Nakaya R. A novel method in the induction of reliable experimental acute and chronic ulcerative colitis in mice. Gastroenterology (1990) 98(3):694–702. doi: 10.1016/0016-5085(90)90290-H 1688816

[20] Chassaing B Aitken JD Malleshappa M Vijay-Kumar M. Dextran sulfate sodium (DSS)-induced colitis in mice. Curr Protoc Immunol (2014) 104:15 25 1–15 25 14. doi: 10.1002/0471142735.im1525s104 PMC3980572 24510619

[21] De Robertis M Massi E Poeta ML Carotti S Morini S Cecchetelli L. The AOM/DSS murine model for the study of colon carcinogenesis: from pathways to diagnosis and therapy studies. J Carcinog (2011) 10:9. doi: 10.4103/1477-3163.78279 21483655 PMC3072657

[22] Baumgart S Chen NM Siveke JT Konig A Zhang JS Singh SK. Inflammation-induced NFATc1-STAT3 transcription complex promotes pancreatic cancer initiation by KrasG12D. Cancer Discovery (2014) 4(6):688–701. doi: 10.1158/2159-8290.CD-13-0593 24694735 PMC4069603

[23] Zhang DJ Wang Q Wei J Baimukanova G Buchholz F Stewart AF. Selective expression of the cre recombinase in late-stage thymocytes using the distal promoter of the lck gene. J Immunol (2005) 174(11):6725–31. doi: 10.4049/jimmunol.174.11.6725 15905512

[24] Neufert C Becker C Neurath MF. An inducible mouse model of colon carcinogenesis for the analysis of sporadic and inflammation-driven tumor progression. Nat Protoc (2007) 2(8):1998–2004. doi: 10.1038/nprot.2007.279 17703211

[25] Maeshima K Yamaoka K Kubo S Nakano K Iwata S Saito K. The JAK inhibitor tofacitinib regulates synovitis through inhibition of interferon-gamma and interleukin-17 production by human CD4+ T cells. Arthritis Rheumatol (2012) 64(6):1790–8. doi: 10.1002/art.34329 22147632

[26] De Vries LCS Duarte JM De Krijger M Welting O Van Hamersveld PHP Van Leeuwen-Hilbers FWM. A JAK1 selective kinase inhibitor and tofacitinib affect macrophage activation and function. Inflammation Bowel Dis (2019) 25(4):647–60. doi: 10.1093/ibd/izy364 30668755

[27] Moolenbeek C Ruitenberg EJ. The "Swiss roll": a simple technique for histological studies of the rodent intestine. Lab Anim (1981) 15(1):57–9. doi: 10.1258/002367781780958577 7022018

[28] Erben U Loddenkemper C Doerfel K Spieckermann S Haller D Heimesaat MM. A guide to histomorphological evaluation of intestinal inflammation in mouse models. Int J Clin Exp Pathol (2014) 7(8):4557–76. Available at: https://www.ncbi.nlm.nih.gov/pubmed/25197329. PMC4152019 25197329

[29] Saito Y Hinoi T Adachi T Miguchi M Niitsu H Kochi M. Synbiotics suppress colitis-induced tumorigenesis in a colon-specific cancer mouse model. PloS One (2019) 14(6):e0216393. doi: 10.1371/journal.pone.0216393 31242213 PMC6594584

[30] Serfling E Avots A Klein-Hessling S Rudolf R Vaeth M Berberich-Siebelt F. NFATc1/alphaA: the other face of NFAT factors in lymphocytes. Cell Commun Signal (2012) 10(1):16. doi: 10.1186/1478-811X-10-16 22764736 PMC3464794

[31] Lagunas L Clipstone NA. Deregulated NFATc1 activity transforms murine fibroblasts *via* an autocrine growth factor-mediated Stat3-dependent pathway. J Cell Biochem (2009) 108(1):237–48. doi: 10.1002/jcb.22245 19565565

[32] Tripathi P Wang Y Coussens M Manda KR Casey AM Lin C. Activation of NFAT signaling establishes a tumorigenic microenvironment through cell autonomous and non-cell autonomous mechanisms. Oncogene (2014) 33(14):1840–9. doi: 10.1038/onc.2013.132 PMC3770739 23624921

[33] Pan M Winslow MM Chen L Kuo A Felsher D Crabtree GR. Enhanced NFATc1 nuclear occupancy causes T cell activation independent of CD28 costimulation. J Immunol (2007) 178(7):4315–21. doi: 10.4049/jimmunol.178.7.4315 17371988

[34] Man K Gabriel SS Liao Y Gloury R Preston S Henstridge DC. Transcription factor IRF4 promotes CD8(+) T cell exhaustion and limits the development of memory-like T cells during chronic infection. Immunity (2017) 47(6):1129–41.e5. doi: 10.1016/j.immuni.2017.11.021 29246443

[35] Dudakov JA Hanash AM van den Brink MR. Interleukin-22: immunobiology and pathology. Annu Rev Immunol (2015) 33:747–85. doi: 10.1146/annurev-immunol-032414-112123 PMC4407497 25706098

[36] Imrich S Hachmeister M Gires O. EpCAM and its potential role in tumor-initiating cells. Cell Adh Migr (2012) 6(1):30–8. doi: 10.4161/cam.18953 PMC3364135 22647938

[37] Trzpis M McLaughlin PM de Leij LM Harmsen MC. Epithelial cell adhesion molecule: more than a carcinoma marker and adhesion molecule. Am J Pathol (2007) 171(2):386–95. doi: 10.2353/ajpath.2007.070152 PMC1934518 17600130

[38] Gerlach K Popp V Wirtz S Al-Saifi R Gonzalez Acera M Atreya R. PU.1-driven Th9 cells promote colorectal cancer in experimental colitis models through il-6 effects in intestinal epithelial cells. J Crohns Colitis (2022) 16(12):1893–910. doi: 10.1093/ecco-jcc/jjac097 PMC10197880 35793807

[39] Kahan BD. Individuality: the barrier to optimal immunosuppression. Nat Rev Immunol (2003) 3(10):831–8. doi: 10.1038/nri1204 14523389

[40] Falvo JV Uglialoro AM Brinkman BM Merika M Parekh BS Tsai EY. Stimulus-specific assembly of enhancer complexes on the tumor necrosis factor alpha gene promoter. Mol Cell Biol (2000) 20(6):2239–47. doi: 10.1128/MCB.20.6.2239-2247.2000 PMC110840 10688670

[41] Jasenosky LD Nambu A Tsytsykova AV Ranjbar S Haridas V Kruidenier L. Identification of a distal locus enhancer element that controls cell type-specific TNF and LTA gene expression in human T cells. J Immunol (2020) 205(9):2479–88. doi: 10.4049/jimmunol.1901311 PMC7576113 32978279

[42] Serfling E Avots A Neumann M. The architecture of the interleukin-2 promoter: a reflection of T lymphocyte activation. Biochim Biophys Acta (1995) 1263(3):181–200. doi: 10.1016/0167-4781(95)00112-T 7548205

[43] Sadlack B Merz H Schorle H Schimpl A Feller AC Horak I. Ulcerative colitis-like disease in mice with a disrupted interleukin-2 gene. Cell (1993) 75(2):253–61. doi: 10.1016/0092-8674(93)80067-O 8402910

[44] Jones DM Read KA Oestreich KJ. Dynamic roles for IL-2-STAT5 signaling in effector and regulatory CD4(+) T cell populations. J Immunol (2020) 205(7):1721–30. doi: 10.4049/jimmunol.2000612 PMC7513451 32958706

[45] Boyman O Sprent J. The role of interleukin-2 during homeostasis and activation of the immune system. Nat Rev Immunol (2012) 12(3):180–90. doi: 10.1038/nri3156 22343569

[46] Sakaguchi S Sakaguchi N Asano M Itoh M Toda M. Immunologic self-tolerance maintained by activated T cells expressing IL-2 receptor alpha-chains (CD25). breakdown of a single mechanism of self-tolerance causes various autoimmune diseases. J Immunol (1995) 155(3):1151–64. Available at: https://www.ncbi.nlm.nih.gov/pubmed/7636184. 7636184

[47] Huehn J Polansky JK Hamann A. Epigenetic control of FOXP3 expression: the key to a stable regulatory T-cell lineage? Nat Rev Immunol (2009) 9(2):83–9. doi: 10.1038/nri2474 19114986

[48] Xiao Y Qureischi M Dietz L Vaeth M Vallabhapurapu SD Klein-Hessling S. Lack of NFATc1 SUMOylation prevents autoimmunity and alloreactivity. J Exp Med (2021) 218(1):1–22. doi: 10.1084/jem.20181853 PMC7953626 32986812

[49] Fan MY Low JS Tanimine N Finn KK Priyadharshini B Germana SK. Differential roles of IL-2 signaling in developing versus mature tregs. Cell Rep (2018) 25(5):1204–13.e4. doi: 10.1016/j.celrep.2018.10.002 30380412 PMC6289175

[50] Govindaraj C Scalzo-Inguanti K Scholzen A Li S Plebanski M. TNFR2 expression on CD25(hi)FOXP3(+) T cells induced upon TCR stimulation of CD4 T cells identifies maximal cytokine-producing effectors. Front Immunol (2013) 4:233. doi: 10.3389/fimmu.2013.00233 23964278 PMC3734366

[51] Wajant H Beilhack A. Targeting regulatory T cells by addressing tumor necrosis factor and its receptors in allogeneic hematopoietic cell transplantation and cancer. Front Immunol (2019) 10:2040. doi: 10.3389/fimmu.2019.02040 31555271 PMC6724557

[52] Fantini MC Rizzo A Fina D Caruso R Sarra M Stolfi C. Smad7 controls resistance of colitogenic T cells to regulatory T cell-mediated suppression. Gastroenterology (2009) 136(4):1308–16.e1-3. doi: 10.1053/j.gastro.2008.12.053 19192480

[53] Saraiva M O'Garra A. The regulation of IL-10 production by immune cells. Nat Rev Immunol (2010) 10(3):170–81. doi: 10.1038/nri2711 20154735

[54] Maynard CL Harrington LE Janowski KM Oliver JR Zindl CL Rudensky AY. Regulatory T cells expressing interleukin 10 develop from Foxp3+ and Foxp3- precursor cells in the absence of interleukin 10. Nat Immunol (2007) 8(9):931–41. doi: 10.1038/ni1504 17694059

[55] Kitani A Fuss I Nakamura K Kumaki F Usui T Strober W. Transforming growth factor (TGF)-beta1-producing regulatory T cells induce smad-mediated interleukin 10 secretion that facilitates coordinated immunoregulatory activity and amelioration of TGF-beta1-mediated fibrosis. J Exp Med (2003) 198(8):1179–88. doi: 10.1084/jem.20030917 PMC2194234 14557415

[56] Sato Y Takahashi S Kinouchi Y Shiraki M Endo K Matsumura Y. IL-10 deficiency leads to somatic mutations in a model of IBD. Carcinogenesis (2006) 27(5):1068–73. doi: 10.1093/carcin/bgi327 16407368

[57] Kuhn R Lohler J Rennick D Rajewsky K Muller W. Interleukin-10-deficient mice develop chronic enterocolitis. Cell (1993) 75(2):263–74. doi: 10.1016/0092-8674(93)80068-P 8402911

[58] Li MC He SH. IL-10 and its related cytokines for treatment of inflammatory bowel disease. World J Gastroenterol (2004) 10(5):620–5. doi: 10.3748/wjg.v10.i5.620 PMC4716896 14991925

[59] Keir M Yi Y Lu T Ghilardi N. The role of IL-22 in intestinal health and disease. J Exp Med (2020) 217(3):e20192195. doi: 10.1084/jem.20192195 32997932 PMC7062536

[60] Sugimoto K Ogawa A Mizoguchi E Shimomura Y Andoh A Bhan AK. IL-22 ameliorates intestinal inflammation in a mouse model of ulcerative colitis. J Clin Invest (2008) 118(2):534–44. doi: 10.1172/JCI33194 PMC2157567 18172556

[61] Wirtz S Popp V Kindermann M Gerlach K Weigmann B Fichtner-Feigl S. Chemically induced mouse models of acute and chronic intestinal inflammation. Nat Protoc (2017) 12(7):1295–309. doi: 10.1038/nprot.2017.044 28569761

