# Supplementary material for: Delayed and limited administration of the JAKinib tofacitinib mitigates chronic DSS-induced colitis
Source: Front Immunol. 2023 May 19;14:1179311. doi: 10.3389/fimmu.2023.1179311 (PMC10235777; doi:10.3389/fimmu.2023.1179311)
Supplement: Supplementary file 1 [file DataSheet_1.pdf]

frontiers

Supplementary Material

Delayed and limited administration of the JAKinib tofacitinib mitigates chronic DSS-induced colitis

Rishav Seal,[1] Lara S. U. Schwab,[1,4] Cristina M. Chiarolla,[1] Nadine Hundhausen,[1] Georg Heinrich Klose,[1,5] Simone Reu-Hofer,[1] Andreas Rosenwald,[1,3] Johannes Wiest,[2] Friederike Berberich-Siebelt[1,#]

[1]Institute of Pathology, Julius-Maximilians-University Wuerzburg, Germany

[2] Institute of Pharmacy and Food Chemistry, Julius-Maximilians-University Wuerzburg, Germany

[3] Comprehensive Cancer Centre Mainfranken, Julius-Maximilians-University Wuerzburg, Germany

[#] **Correspondence:**
Friederike Berberich-Siebelt
path230@mail.uni-wuerzburg.de

[4] Present address: Department of Microbiology and Immunology, Peter Doherty Institute for Infection and Immunity, The University of Melbourne, Victoria, Australia. / Institute of Clinical Chemistry and Clinical Pharmacology, University Hospital Bonn, Bonn, Germany.

[5] Present address: Institute of Pathology, University Hospital Carl Gustav Carus, Medical Faculty, Technical University Dresden, Dresden, Germany.

Supplementary Table 1: Tofacitinib physical chemical properties

Jain, Clinical Pharmacology and Biopharmaceutics Review, FDA, 2011 203214Orig1s000ClinPharmR.pdf (fda.gov)

Molecular Formula	$C_{16}H_{2}0N_{6}O{\bullet}C_{6}H_{8}O_{7}$
Molecular Weight	504.5 g/mol (312.4 g/mol as free base)
Physical State	Powder
Polymorphism	There is only CP-690,550-10
Dissociation constant	pK_a = 5.07
Solubility	- Water: 2.9 mg/ml (freely soluble in water)<br>- 3.48 – >28 mg/(ml in aqueous solution of pH 1 – 3.9<br>- 0.20 – 0.59 mg/ml in aqueous solution of pH 4.53 – >8<br>- Solubility decreases with increase in pH
Partition Coefficient	Log P = 1.15 of the neutral form (free base)<br>Average partition coefficient = 14.3 at pH 7.3

Supplementary Table 2: Rational of Tofacitinib concentration in drinking water

Documented dosing	- Ghoreschi et al, 2011: 2 x 50 mg/kg/d orally<br>- Maeshima et al., 2012: 1.5 mg/kg/d – 15 mg/kg/d subcutaneously
Dose per mouse (≤25 mg)	- Ghoreschi et al., 2011: 2.5 mg per day<br>- Maeshima et al., 2012 : 0.0375 / 0.375 mg per day
Mice drinking volume	5 – 6 ml/d
Chosen oral dosing	2.5 mg / 6 ml → 0.42 mg/ml
Addition for taste	+ 0.1 % sucrose

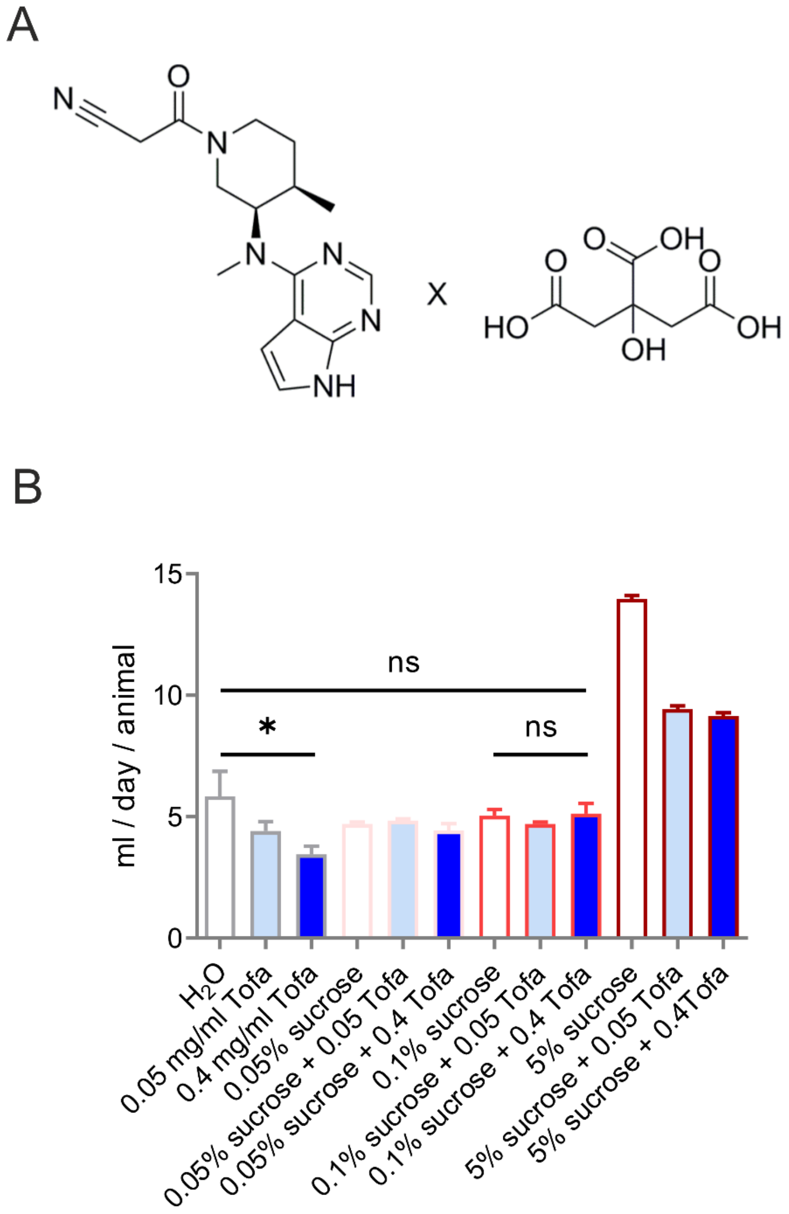

Supplementary Figure 1. Tofacitinib in drinking water is well tolerated, especially with the addition of 0.1 % sucrose. (**A**) Structure of tofacitinib citrate. (**B**) Mice were offered modified drinking water for 7 days, weighed before and after and the group-wise uptake evaluated daily by measuring the differences in liquid amount within the bottles. n= 5 – 10 per group.

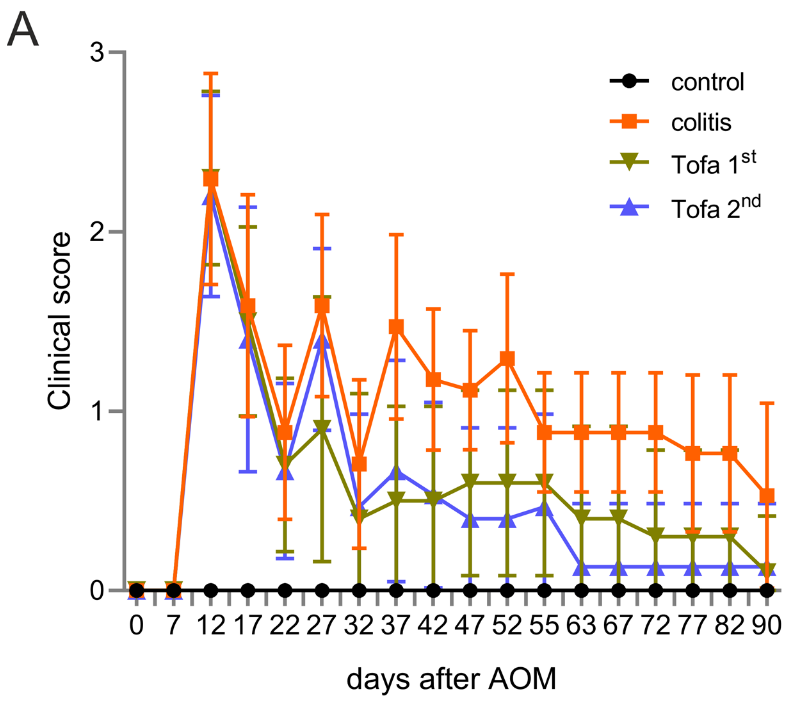

Supplementary Figure 2. The clinical score resolves best after late onset treatment with tofacitinib. (**A**) Clinical score of all groups treated according to the scheme in Figure 2A; mean ± SD. Data representative of three independent experiments; at least n=3 in each group.

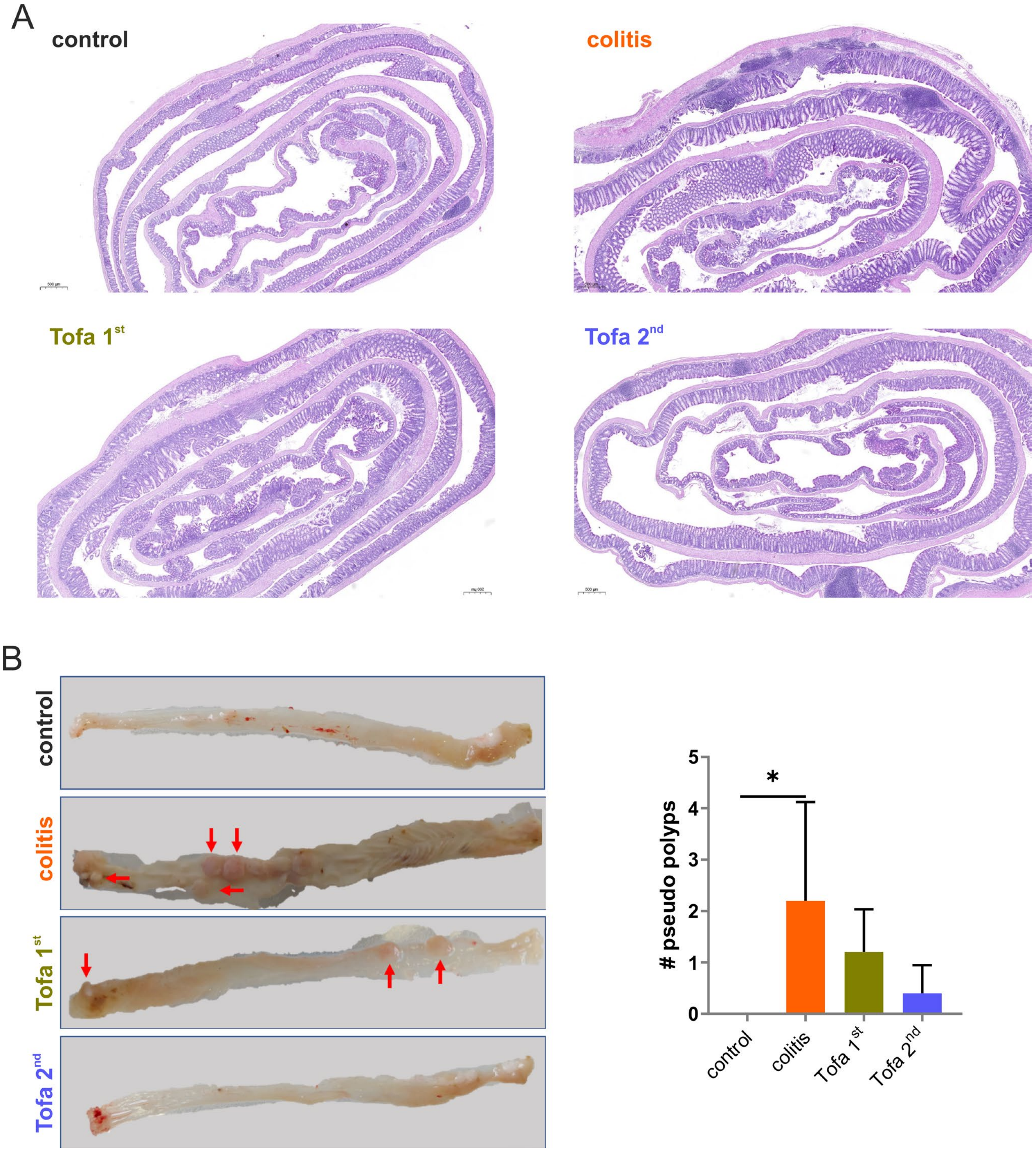

Supplementary Figure 3. Occurrence of pseudo polys is best prevented upon late onset treatment with tofacitinib. (**A**) Corresponding H&E stainings to Figure 3A, but with 20x magnification only. (**B**) Colons (from caecum to rectum) of all treatment groups. Arrows indicate pseudo polyps, n=5. Unpaired Student's t test (*$p \leq 0.05$). Data representative of three independent experiments; at least n=3 in each group.
